# Exploring the Interplay Between Glycated Albumin, AGEs, and Inflammation in Old Patients with CKD

**DOI:** 10.3390/metabo15080515

**Published:** 2025-08-01

**Authors:** Simone Vettoretti, Lara Caldiroli, Paolo Molinari, Amanda Villa, Massimiliano M. Corsi Romanelli, Elena Vianello, Elena Dozio, Simonetta Genovesi

**Affiliations:** 1 Unit of Nephrology and Dialysis Fondazione IRCCS San Gerardo di Monza, 20900 Monza, Italy; simone.vettoretti@unimib.it; 2 School of Medicine and Surgery, University of Milan Bicocca, 20126 Milan, Italy; a.villa148@campus.unimib.it; 3 Unit of Nephrology, Dialysis and Kidney Transplantation Fondazione IRCCS Ca’ Granda Ospedale Maggiore Policlinico di Milano, 20122 Milan, Italy; lara.cardiroli@policlinico.mi.it (L.C.); paolo.molinari@policlinico.mi.it (P.M.); 4 Department of Biomedical Sciences for Health, Università degli Studi di Milano, 20133 Milan, Italy; mmcorsi@unimi.it (M.M.C.R.); elena.vianello@unimi.it (E.V.); elena.dozio@unimi.it (E.D.); 5 Department of Clinical and Experimental Pathology, IRCCS Istituto Auxologico Italiano, 20149 Milan, Italy; 6 Experimental Laboratory for Research on Organ Damage Biomarkers, IRCCS Istituto Auxologico Italiano, 20149 Milan, Italy; 7 Department of Cardiology, IRCCS Istituto Auxologico Italiano, 20149 Milan, Italy

**Keywords:** glycated albumin advanced glycation end-products, inflammation, chronic kidney disease

## Abstract

Introduction: Chronic kidney disease (CKD) increases cardiovascular risk through mechanisms such as oxidative stress and the accumulation of advanced glycation end products (AGEs). Glycated albumin (GA) is associated with cardiovascular risk in CKD patients, but its relationship with AGEs and systemic inflammation remains unclear. This study investigated these associations in old patients with severe CKD, with and without diabetes. Methods: We conducted a cross-sectional analysis in 122 patients aged ≥ 65 years with CKD stages G3a–G5, including 67 diabetics and 55 non-diabetics. Patients with confounding comorbidities were excluded. We measured GA, AGEs, various AGEs receptors (RAGE) isoforms, and inflammatory cytokines (CRP, IL-6, TNFα, and MCP-1) using standardized assays. Statistical analyses included group comparisons, correlation coefficients, and multivariate regression. Results: Of 122 patients (mean age 77.7 ± 11.3 years), diabetics had higher GA percentages than non-diabetics (22.0 ± 7.1% vs. 17.5 ± 5.4%, *p* = 0.0001), while AGEs (2931 ± 763 vs. 3156 ± 809 AU; *p* = 0.118) and inflammatory markers (CRP 0.240[0.380] vs. 0.200[0.280] mg/dL; *p* = 0.142; IL-6 3.4[4.0] vs. 3.0[3.8] pg/mL; *p* = 0.238) were similar between groups. Overall, GA was inversely correlated with estimated glomerular filtration rate (eGFR) (ρ = −0.189, *p* = 0.037) and positively with glycated hemoglobin (HbA1c) (ρ = 0.525, *p* < 0.0001), but showed no significant correlation with AGEs, RAGE isoforms, or inflammatory cytokines. In multivariate analysis, only HbA1c remained independently associated with GA (β = 0.222, *p* = 0.005). Conclusions: In old patients with severe CKD, GA appears to be a more useful marker of glycemic control than glycation stress, the latter of which is the result of multiple factors, including impaired kidney function and systemic inflammation.

## 1. Introduction

Chronic kidney disease (CKD) is a major clinical condition associated with an elevated risk of cardiovascular (CV) events [1]. This increased risk arises from a multifactorial etiology involving both traditional risk factors (e.g., diabetes, hypertension, and dyslipidemia) [1] and non-traditional mechanisms, including chronic systemic inflammation, metabolic disturbances, accumulation of uremic toxins [2,3], and oxidative stress-induced endothelial dysfunction [2,4]. Among the latter, oxidative stress plays a key role in promoting the non-enzymatic glycation of circulating proteins including albumin. Maillard reaction is one of the pathways involved in protein glycation. It begins with the covalent attachment of reducing sugars (e.g., glucose) to free amino groups on proteins. This reaction progresses through three stages: formation of a reversible Schiff base (aldimine), rearrangement into a more stable Amadori product (ketoamine), and subsequent chemical transformations, such as rearrangement, oxidation, polymerization, and fragmentation of early glycation products, that generate irreversible advanced glycation end products (AGEs) [5,6,7].

Glycated albumin (GA) accounts for approximately 80% of circulating ketoamines (fructosamine) and is recognized as the principal early glycation product (EGP) in plasma [8]. In physiological conditions, AGEs are primarily catabolized in the proximal renal tubules and excreted into the urine [9,10].

However, in CKD, compromised glomerular filtration, tubular dysfunction, and oxidative stress lead to systemic AGE accumulation, which further exacerbates inflammation, endothelial damage, and tissue fibrosis. These effects contribute to both renal disease progression and increased cardiovascular risk [11]. Thus, CKD serves as a prototypical state of enhanced protein glycation, driven by reduced renal clearance and a systemic pro-oxidant, pro-inflammatory milieu. In CKD, elevated GA levels also reflect persistent hyperglycemia and are increasingly recognized as indicators of oxidative stress and systemic inflammation [12].

Among diabetic patients with CKD, GA emerged as a superior alternative biomarker for evaluating glycemic control. It reflects mean blood glucose levels over the previous 2–3 weeks, thereby providing a more immediate measure of glycemic variability compared to glycated hemoglobin (HbA1c). Unlike HbA1c, GA is not affected by changes in erythrocyte turnover, altered red blood cell lifespan, or erythropoietin therapy, making it particularly useful in patients with advanced CKD or those undergoing hemodialysis [13]. In addition, as a precursor of AGEs, GA plays a central role in initiating downstream pathophysiological processes that culminate into AGE formation. AGEs exert harmful biological effects via two main mechanisms: (i) alteration of protein structure and function, and (ii) activation of intracellular signaling pathways through binding to receptors such as the receptor for advanced glycation end products (RAGE). Engagement of RAGE leads to the generation of reactive oxygen species (ROS), amplification of inflammatory signaling, and increased leukocyte adhesion [7,14,15]. GA itself possesses direct pro-inflammatory activity. It has been shown to upregulate the expression of interleukin-8 (IL-8) and intercellular adhesion molecule-1 (ICAM-1) in renal tubular cells and to activate the NF-κB signaling pathway through RAGE binding [16]. This activation promotes the transcription of several pro-inflammatory (TNFα, IL-6, IL-2β, and CCL 2) [17] and pro-fibrotic mediators, including connective tissue growth factor (CTGF), transforming growth factor-beta (TGF-β), and vascular endothelial growth factor (VEGF) [16], which can further promote kidney fibrosis.

In the present study, we investigated the associations between GA, AGEs, and markers of systemic inflammation in older patients with advanced CKD, with and without diabetes mellitus. Our aim was to assess whether renal impairment may have influenced the association of GA with AGEs and systemic inflammation in these patients.

## 2. Methods

### 2.1. Patients and Study Design

We evaluated cross-sectionally 122 prevalent patients that were enrolled according to the following criteria: age ≥ 65 years, CKD stages G3a to G5 in conservative therapy, and with a relatively stable glomerular filtration rate (GFR) over the previous 6 months (with less than 2 mL/min/1.73/m^2^ of variation). GFR was estimated according to the CKD-EPI formula 2021 (eGFR). We excluded patients with cancer, cirrhosis and/or ascites, severe heart failure (NYHA class III–IV), nephrotic and or nephritic syndrome, thyroid diseases, bowel inflammatory diseases, decompensated diabetes (i.e., if HbA1C > 86 mmol/mol), and inability to cooperate. We also excluded patients treated with immunosuppressive drugs or who had been hospitalized in the last three months. Twenty-four-hour urinary collection and biochemical parameters were collected the day of the visit after overnight fasting of at least 12 h. The study was conducted according to the ICP Good Clinical Practices Guidelines, and it was approved by the ethics committee of our institution (Milano2-approval N. 347/2010). All patients signed an informed consent to participate.

### 2.2. Glycated Albumin Assay

Plasma glycated albumin (GA) and percentage of glycated albumin (GA%) were determined using the enzymatic QuantiLab^®^ Glycated Albumin assay (Instrumentation Laboratory S.p.A., Milan, Italy) on an ILab650 system (Instrumentation LaboratoryS.p.A., Milan, Italy—a Werfen Company S.p.A, Milan, Italy). The lab analyzer automatically calculated the results for each sample. GA% was calculated as the ratio between GA and albumin, with an arithmetic algorithm applied to align GA% levels with those obtained by the HPLC reference method [18,19,20]. The minimum detectable concentration for GA was 1.15 g/L. The maximum intra- and inter-assay coefficients of variation were 2.1% and 1.3% for GA, respectively, and 1.2% and 1.0% for GA%, respectively.

### 2.3. AGEs Quantification

We quantified plasma AGEs using a fluorometric method, as previously described [21,22,23]. Briefly, 100 μL of each plasma sample was added to a 96-well black microplate. Fluorescence intensity was then measured at 414–445 nm after excitation at 365 nm using a GloMax^®^-Multi Microplate Multi-mode Reader (Promega, Milan, Italy). The fluorescence signal intensity was expressed in arbitrary units (AUs). Finally, the AGE content was normalized to the total serum protein content. The average inter- and intra-assay coefficients of variation (CV) for fluorescent AGEs were 7.3% and 5.99%, respectively.

### 2.4. Quantification of sRAGE, esRAGE and cRAGE

The quantification of total RAGE isoforms was performed as previously described [15]. Briefly, soluble RAGE (sRAGE) and endogenous secretory RAGE (esRAGE) were measured using two distinct ELISA kits: the R&D Systems kit (DY1145, Minneapolis, MN, USA) for sRAGE, and the B-Bridged International kit (K1009–1, Santa Clara, CA, USA) for esRAGE. For the esRAGE assay, the intra- and inter-assay coefficients of variation were 6.37% and 4.78–8.97%, respectively. Cleaved RAGE (cRAGE) levels were then calculated by subtracting esRAGE from totals RAGE. Finally, the AGE/sRAGE ratio was determined. All photometric measurements were conducted using a GloMax^®^-Multi Microplate Multimode Reader (Promega, Milan, Italy).

### 2.5. Interleukins Quantification

Serum samples for the determination of cytokine concentration were collected on the day of the visit, then they were centrifuged for 10 min at 3500× *g* rpm to separate serum and plasma and stored immediately at −80 °C. All samples were thawed at most three times. Cytokines concentrations were performed by using enzyme-linked immunosorbent assay (ELISA) kits following the manufacturer’s instructions. The following kits were used: Human IL-10 ELISA Kit EHIL10 (Invitrogen, Thermo Fisher Scientific, Monza, Italy), and the Quantikine ELISA Human CCL2/MCP-1 Immunoassay DCP00 Human TNF-alpha ELISA Kit (Thermo Fisher Scientific, Monza, Italy). For IL-6 dosage, three different ELISA kits, with standard curve ranges of decreasing values, were used and compared: Human IL-6 ELISA Kit EH2IL6 (Thermo Fisher Scientific, Monza, Italy), Human IL-6 Platinum ELISA BMS213/2 (Affymetrix, Thermo Fisher Scientific, Monza, Italy), and Quantikine HS ELISA Human IL-6 Immunoassay HS600B (R&D Systems, Space, Milano, Italy), with sensitivity of <1 pg/m, 0.92 pg/mL and 0.110 pg/mL, respectively. For IL-6 quantification, Quantikine HS ELISA, Human IL-6 Immunoassay HS600B, and Human IL-6 ELISA Kit EH2IL6 results were compared by a simple regression test and both results were indifferently used after ascertaining the significant correlation. Serum levels of MCP1 were measured with a commercially available ELISA kit (R&D Systems, Minneapolis, MN, USA) according to the manufacturer’s instructions. In each test, the curve included the zero as the last standard point. Quantikine Immunoassay Control Group 1–4 or 10 (R&D Systems, Space, Milano, Italy), as appropriate, were used to check the acceptability of the assays. Absorbance readings were measured at 450 nm by spectrophotometer (Xenius Safas, Monaco). All cytokines’ values were evaluated in duplicate.

### 2.6. Statistical Analysis

Continuous variables were expressed as mean ± standard deviation (SD) in parametric distributions, or in the median and interquartile range (IQR) in non-parametric data. Categorical variables were summarized as percentages. Parametric variables were compared with Student’s *t*-test, while we performed the Mann–Whitney “U” test for the comparison of not parametric ones. Proportions and categorical variables were compared using the independent chi-squared (χ^2^) test or the Fisher’s exact test. Correlations were explored using Pearson or Spearman linear regressions for parametric and not parametric variables, respectively. All the variables that were associated with GA at univariate analysis were included in a multivariate regression model, where GA was the dependent variable. Since GA had a skewed distribution, it was log base 10 transformed before being analyzed by multivariate regression. A *p*-value less than 0.05 was considered significant. Statistical analysis was conducted using Statview 5.1.

## 3. Results

### 3.1. General Characteristics of the Population

The demographic and clinical characteristics are described in Table 1. We enrolled 122 participants, including 67 individuals with type 2 diabetes mellitus and 55 non-diabetic controls. The mean age of the overall population was 77.7 ± 11.3 years, with no significant difference between diabetic and non-diabetic participants (78.8 ± 7.6 vs. 76.4 ± 14.4 years; *p* = 0.240). Sex distribution was comparable between the two groups (male/female, %: 76/24 vs. 68/32; *p* = 0.309) as the BMI (28.7 ± 5.2 vs. 26.7 ± 4.0 kg/m^2^; *p* = 0.178).

Both groups exhibited reduced kidney function consistent with advanced CKD, and the eGFR was significantly higher in diabetic patients compared to non-diabetic ones (27 ± 11 vs. 22 ± 10 mL/min/m^2^; *p* = 0.008).

No significant differences were observed for proteinuria (600 (1569) vs. 435 (990) mg/24 h; *p* = 0.301) serum albumin (4.03 ± 0.37 vs. 4.07 ± 0.34 g/dL; *p* = 0.457), total cholesterol (165 ± 35 vs. 170 ± 37 mg/dL; *p* = 0.483), HDL cholesterol (52 ± 15 vs. 55 ± 21 mg/dL; *p* = 0.324), or triglycerides (133 ± 56 vs. 118 ± 50 mg/dL; *p* = 0.126).

As expected, the glycated hemoglobin (HbA1c) levels were markedly higher in diabetic group (51.5 ± 10.8 vs. 38.3 ± 4.3 mmol/mol; *p* < 0.0001).

Inflammatory markers, including C-reactive protein (CRP: 0.240 (0.380) vs. 0.200 (0.280) mg/dL; *p* = 0.142), tumor necrosis factor-alpha (TNFα: 13.9 (10.1) vs. 13.7 (10.1) pg/mL; *p* = 0.660), interleukin-6 (IL-6: 3.4(4.0) vs. 3.0 (3.8) pg/mL; *p* = 0.238), and monocyte chemotactic protein-1 (MCP-1: 403 (207) vs. 408 (241) pg/mL; *p* = 0.884), did not differ significantly between groups.

### 3.2. Products of Glycation and Their Receptors

Table 2 depicts the comparison of serum levels of the different products of glycation in the overall population and in diabetic and non-diabetic subgroups. Serum glycated albumin (GA) concentrations were significantly elevated in diabetic individuals compared to non-diabetics (22.0 ± 7.1% vs. 17.5 ± 5.4%; *p* = 0.0001). Conversely, no significant differences were observed between groups in circulating levels of advanced glycation end products (AGEs: 2931 ± 763 vs. 3156 ± 809 arbitrary units; *p* = 0.118), soluble RAGEs (sRAGEs: 1876 [1178] vs. 1935 [1912] pg/mL; *p* = 0.316), endogenous secretory RAGEs (esRAGEs: 527 [346] vs. 562 [387] pg/mL; and *p* = 0.204 or cleaved RAGEs (cRAGEs: 1300 [786] vs. 1347 [1375] pg/mL; *p* = 0.341).

### 3.3. Correlations of Glycated Albumin

The correlation analysis of GA with relevant biochemical parameters, glycation products, and inflammatory markers is presented in Table 3. In the overall population, GA showed a significant inverse correlation with eGFR (ρ = −0.189; *p* = 0.037), whereas no correlation was found with 24 h proteinuria (ρ = 0.056; *p* = 0.769); no statistical differences were found in subgroup analyses between diabetic and non-diabetic patients. GA correlated positively with HbA1c in the overall population (ρ = 0.525; *p* < 0.0001), but in subgroup analysis, the correlation was maintained in diabetics (ρ = 0.434; *p* = 0.0008), but not in non-diabetics (ρ = 0.174; *p* = 0.377). GA did not have any correlation with serum albumin in the overall population (ρ = −0.031, *p* = 0.735) or in subgroups as well (diabetics: ρ = −0.146; *p* = 0.241; not diabetics: ρ = 0.194; *p* = 0.154). In the overall population, GA did not have any correlation with glycation products AGEs (ρ = 0.028; *p* = 0.535), sRAGEs (ρ = −0.074; *p* = 0.418), cRAGEs (ρ = −0.059; *p* = 0.518), or esRAGEs (ρ = −0.130; *p* = 0.155), and these associations persisted as being not significant across both diabetic and non-diabetic subgroups. Lastly, GA did not significantly correlate with any of the inflammatory markers, including CRP (ρ = 0.072; *p* = 0.427), TNF-α (ρ = −0.060; *p* = 0.531), IL-6 (ρ = −0.048; *p* = 0.614), and MCP-1 (ρ = −0.072; *p* = 0.489); similar results were observed in subgroup analyses. When we evaluated in a model of multivariate regression analysis (Table 4) the influence of the variables that were associated with GA at univariate analysis (i.e., eGFR and HbA1c), the overall model predicted 8% of GA variability, but only HbA1c maintained an independent correlation with GA.

## 4. Discussion

The main result of our exploratory evaluation is that, within a population of patients with advanced CKD, GA exhibits no correlation with AGEs or systemic inflammation. Notably, this lack of correlation persists even when patients have been stratified into subgroups with and without diabetes. The absence of a correlation between GA and AGEs was unexpected, given that they are presumed to share common pathophysiological pathways [6,7,8,9]. Although numerous studies support a correlation between GA and AGEs, particularly in patients with diabetes mellitus [8,13,24], other specific clinical conditions, such as CKD, may influence this relationship. In patients with CKD, increased oxidative stress and a decreased glomerular filtration rate can affect the production and accumulation of advanced glycation end products (AGEs). We assume that, in this context, the absence of a correlation should be examined in relation to the role of renal dysfunction in regulating the overproduction and accumulation of AGEs [14]. This could potentially occur independently of the pathways typically involved in albumin glycation [25], despite the overall pro-inflammatory and pro-oxidant milieu. This hypothesis is partly supported by the observation that AGEs and GA correlate with GFR in opposing ways within our population. Specifically, while GA increases with increasing GFR (Table 3), AGEs tend to decrease (Figure S1). Furthermore, at lower levels of GFR, both diabetic and non-diabetic patients exhibit similar AGEs concentrations (Figure S1), suggesting that in CKD patients, AGEs are more closely associated with eGFR than with glycemic control. In our opinion, there are two key mechanisms that can drive these discrepancies. Firstly, the reduced kidney filtration strongly promoted the accumulation of molecules that are normally filtrated, such as AGEs. Secondarily, malnutrition and inflammation, which are associated with reduced renal function [26,27,28] and increased albumin turnover [29,30], can contribute to reduce albumin glycation rate. In our population, GA does not correlate with albumin levels. This finding may be due to the fact that albumin levels in the CKD population are influenced by several variables, such as the degree of proteinuria, systemic inflammatory state, and nutritional status. Regarding the first factor, our analysis reveals no association between GA and 24 h proteinuria. Furthermore, GA shows no association with key inflammatory markers. However, this does not mean it is impossible for these markers to be differentially correlated with GA and nutritional status. Indeed, it is possible that the increased inflammatory state observed in elderly patients with CKD [31] may differentially influence GA levels [24,25] and overall albumin turnover [30,32]. The lack of correlation between GA and inflammatory markers may also depend on the fact that we selected on purpose a bench of cytokines that are increased at lower GFR [31], while GA is correlated with GFR in the opposite manner; therefore, kidney dysfunction may have influenced the interaction between GA and inflammatory markers.

Considering that GA is very sensitive to the hyperglycemic state, the higher levels observed in diabetic in comparison to non-diabetic CKD patients can be just due to this metabolic alteration [24]. This possibility is supported by the results of the multivariate analysis that suggest HbA1c being the only independent factor associated with GA levels.

Glycated albumin levels tend to be higher in people with diabetes than in those without. Currently, there is not a standardized cut-off value for GA in healthy individuals. However, according to published research on reference ranges in the healthy Italian population [33,34], it seems that patients with CKD, both diabetic and non-diabetic, tend to have higher GA levels than healthy controls. In non-diabetic patients, this elevation is largely attributed to the increased pro-oxidant state that characterizes their condition and can accelerate protein glycation. Notably, both in the overall population as well as in the subgroups with and without diabetes, GA was associated with glycated hemoglobin, but not with AGEs. AGEs were also not correlated with glycated hemoglobin nor with glycemia. Although AGE production can be also affected by glucose levels and the duration of hyperglycemia [35,36], in CKD, their levels seem to be mainly affected by systemic inflammation [37,38] and reduced glomerular filtration rate [39,40,41]. These observations may explain why, in our study, non-diabetic patients with lower GFR values exhibit AGE levels similar to those of diabetic patients. In the overall population as well as in both the subgroups, we did not find any association between GA and RAGEs. We believe that as for AGEs, this may depend on the opposite correlation of RAGEs and GFR [41], beyond the fact that RAGEs are strictly associated with AGEs production and accumulation [42,43].

Our study offers a novel perspective on this topic. There are just a few data about AGEs and sRAGE in non-dialysis patients, and most of them have been recently published by our group. It is crucial to highlight that DM is only one of many causes of CKD. In our research, we specifically included patients with CKD stemming from diverse etiologies, not just DM. We previously published findings indicating that in advanced CKD, AGEs levels do not significantly differ between DM and non-DM patients. This is not surprising, as numerous factors beyond DM can drive AGE accumulation in these individuals, as thoroughly reviewed by our group [14], including oxidative stress and reduced kidney filtration. As already stated in the previous sections, the lack of correlation between product of glycation and sRAGE is different from data published in other manuscripts [44], but it can be explained by considering at least three key aspects. First, there is a complex interplay of factors in advanced CKD that differentially modulates the synthesis and clearance of AGEs and sRAGE. It is particularly important to note that sRAGE is a heterogeneous pool, consisting of both the cleaved form of membrane-bound RAGE (cRAGE) and the alternative splicing form (esRAGE). The levels of these two sRAGE molecules can be distinctly affected by stimuli such as oxidative stress, RAGE activation, and kidney filtration [14]. Therefore, mechanisms beyond DM itself likely promote AGE accumulation and disrupt the expected correlation within the AGE-RAGE system in CKD. Second, our AGEs evaluation relied on a fluorometric method, primarily focusing on fluorescent AGEs. Finally, considering that sRAGE levels can be modulated by specific pharmacological treatments [45], the correlations reported across various studies may also reflect such drug-related confounding factors.

Our study has some limitations. First, we evaluated a relatively small number of patients. Secondly, we included only patients with severe CKD; therefore, we may have missed the correlations of the considered variables in the early stages of CKD. In particular, given that AGE accumulation is strongly associated with eGFR decline, our findings on the relationship between GA and AGEs may differ in the early stages of CKD. Finally, we did not assess specific nutritional parameters that could have further clarified potential correlations between GA, inflammation, and malnutrition.

Conversely, our study also has several strengths. The population enrolled was selected to include only stable patients, thereby excluding comorbidities that could have influenced the correlation between GA, AGEs, and systemic inflammation independently. We included in our analysis both AGEs and various isoforms of RAGEs, updating the knowledge regarding possible associations between these elements in patients with advanced CKD with and without diabetes. Furthermore, we explored potential associations between GA and systemic inflammation by assessing interleukins that are recognized to be increased in CKD patients [31].

## 5. Conclusions

Our results indicate that in old patients with advanced CKD, plasma GA levels do not correlate with AGEs, RAGEs, or systemic inflammation, regardless of the presence or absence of diabetes. Therefore, in CKD, GA appears to be more indicative of glycemic control than of glycation stress, which results from multiple factors, including impaired kidney function and systemic inflammation.

## Figures and Tables

**Table 1:** Population characteristics.

	Overall Population	Diabetics	Non-Diabetics	*p*-Value *
	n = 122	n = 67	n = 55	
**General characteristics**				
Age, years	77.7 ± 11.3	78.8 ± 7.6	76.4 ± 14.4	0.240
Sex (m/f,%)	72/28	76/24	68/32	0.309
BMI, Kg/m^2^	27.8 ± 4.7	28.7 ± 5.2	26.7 ± 4.0	0.178
eGFR, mL/min/1.73 m^2^	24 ± 11	27 ± 11	22 ± 10	0.008
Proteinuria, mg/24 h	525 (1049)	600 (1569)	435 (990)	0.301
Serum albumin, g/dL	4.05 ± 0.35	4.03 ± 0.37	4.07 ± 0.34	0.457
HbA1c, mmol/mol	47.2 ± 11.1	51.5 ± 10.8	38.3 ± 4.3	<0.0001
Glycemia, mg/dL	116 ± 38	137 ± 39	91 ± 10	0.0084
Total cholesterol, mg/dL	168 ± 36	165 ± 35	170 ± 37	0.483
HDL cholesterol, mg/dL	53 ± 18	52 ± 15	55 ± 21	0.324
Triglycerides, mg/dL	126 ± 54	133 ± 56	118 ± 50	0.126
**Inflammatory markers**				
CRP, mg/dL	0.215 (0.315)	0.240 (0.380)	0.200 (0.280)	0.142
TNFα, pg/mL	13.7 (10.2)	13.9 (10.1)	13.7 (10.1)	0.660
IL-6, pg/mL	3.1 (4.0)	3.4 (4.0)	3.0 (3.8)	0.238
MCP-1, pg/mL	408 (218)	403 (207)	408 (241)	0.884

BMI: body mass index; eGFR: estimated glomerular filtration rate; HbA1c: glycated hemoglobin; CRP: C reactive protein; TNFα tumor necrosis factor; IL-6: interleukin 6; MCP-1 macrophage chemotactic protein 1; TNFα tumor necrosis factor alfa; IL-6: interleukin 6; MCP-1 macrophage chemotactic protein 1; and * *p* for diabetics vs. non-diabetics; data are expressed as mean ± SD or median (IQR).

**Table 2:** Products of glycation and their receptors.

	Overall Population	Diabetics	Non-Diabetics	*p*-Value *
GA, %	19.7 (6.2)	22.0 (7.1)	17.5 (5.4)	0.0001
AGEs, arbitrary units	3032 ± 789	2931 ± 763	3156 ± 809	0.118
sRAGEs, pg/mL	1895 (1370)	1876 (1178)	1935 (1912)	0.316
esRAGEs, pg/mL	542 (354)	527 (346)	562 (387)	0.204
cRAGEs, pg/mL	1341 (1048)	1300 (786)	1347 (1375)	0.341

GA: glycated albumin; AGEs: advanced glycation end products; and RAGEs: receptor of the advanced glycated end products. Data are expressed as mean ± SD or median (IQR) * *p* for diabetics vs. non-diabetics.

**Table 3:** Correlations of GA with kidney function, metabolic parameters, AGEs, RAGEs, and inflammatory markers.

	Overall Population	Diabetics	Non-Diabetics
**Kidney function**			
GA vs. eGFR	ρ = 0.189; *p* = 0.037	ρ = 0.107; *p* = 0.385	ρ = 0.043; *p* = 0.752
GA vs. Proteinuria	ρ = 0.056; *p* = 0.769	ρ = −0.004; *p* = 0.978	ρ = −0.104; *p* = 0.418
**Metabolic parameters**			
GA vs. HbA1c	ρ = 0.525; *p* ≤ 0.0001	ρ = 0.434; *p* = 0.0008	ρ = 0.174; *p* = 0.377
GA vs. serum albumin	ρ = −0.031; *p* = 0.735	ρ = −0.146; *p* = 0.241	ρ = 0.194; *p* = 0.154
**Glycosilation products**			
GA vs. AGEs	ρ = 0.028; *p* = 0.535	ρ = 0.144; *p* = 0.243	ρ = 0.185; *p* = 0.174
GA vs. sRAGES	ρ = −0.074; *p* = 0.418	ρ = 0.101; *p* = 0.412	ρ = −0.171; *p* = 0.208
GA vs. cRAGES	ρ = −0.059; *p* = 0.518	ρ = 0.126; *p* = 0.310	ρ = −0.167; *p* = 0.219
GA vs. esRAGES	ρ = −0.130; *p* = 0.155	ρ = −0.031; *p* = 0.798	ρ = −0.123; *p* = 0.368
**Inflammatory markers**			
GA vs. CRP	ρ = 0.072; *p* = 0.427	ρ = 0.411; *p* = 0.413	ρ = 0.056; *p* = 0.683
GA vs. TNFα	ρ = −0.060; *p* = 0.531	ρ = 0.260; *p* = 0.838	ρ = −0.272; *p* = 0.600
GA vs. IL-6	ρ = −0.048; *p* = 0.614	ρ = −0.052; *p* = 0.684	ρ = −0.226; *p* = 0.125
GA vs. MCP-1	ρ = −0.072; *p* = 0.489	ρ = −0.009; *p* = 0.946	ρ = −0.196; *p* = 0.247

GA: glycated albumin; HbA1c: glycated hemoglobin; AGEs: advanced glycation end products; sRAGE: soluble receptor of AGES; cRAGE: cleaved receptor of AGEs; esRAGE: endogenous secretory receptor of AGEs; CRP: C reactive protein; TNFα: tumor necrosis factor alfa; IL-6: interleukin 6; and MCP-1: monocyte chemotactic protein-1.

**Table 4:** Multivariate regression analysis of the association between GA, eGFR, and HbA1c.

	Count	R^2^	Adj R^2^	F	*p*-Value
**Overall model**	122	0.101	0.080	4.825	0.010
Regression coefficients					
	**Coeff**	**Std. Error**	**Std Coeff.**	***t*** **-Value**	***p*** **-Value**
Intercept	10.762	3.786	10.762	2.843	0.0056
eGFR	0.034	0.080	0.044	0.420	0.6759
HbA1c	0.222	0.078	0.303	2.861	0.0053

eGFR: estimated glomerular filtration rate; HbA1c: glycated hemoglobin.

## Data Availability

The original datasets analyzed in the current study are not publicly available since they belong to a large dataset that is shared in a research consortium involving Fondazione IRCCS Ca’ Granda Ospedale Maggiore Policlinico di Milano and other Departments of the University of Milan and University of Milan Bicocca. Therefore, some data are currently under analysis also for other research purposes. The original dataset could be provided by the corresponding author, upon the approval of the other members of the consortium, on reasonable request.

## Supplementary Materials

The following supporting information can be downloaded at: https://www.mdpi.com/article/10.3390/metabo15080515/s1, Figure S1: correlation of GFR with AGEs in the overall population and in subgroups with and without diabetes.
