# Supplementary material for: Exploring the Interplay Between Glycated Albumin, AGEs, and Inflammation in Old Patients with CKD
Source: Metabolites. 2025 Aug 1;15(8):515. doi: 10.3390/metabo15080515 (PMC12388635; doi:10.3390/metabo15080515)
Supplement: Supplementary file 1 [file metabolites-15-00515-s001.zip › metabolites-3749943-supplementary.pdf]

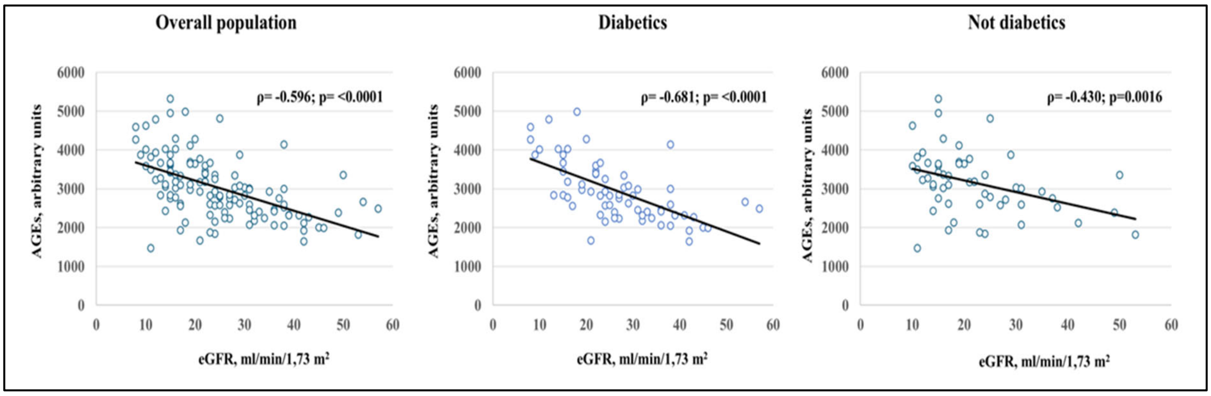

Figure S1: correlation of GFR with AGEs in the overall population and in subgroups with and without diabetes
